# Nutritional Deficiencies and Oral Candidiasis in Children from Northeastern Romania: A Cross-Sectional Biochemical Assessment

**DOI:** 10.3390/nu17111815

**Published:** 2025-05-27

**Authors:** Alexandru-Emilian Flondor, Irina-Georgeta Sufaru, Ioana Martu, Stefan-Lucian Burlea, Catalina Flondor, Vasilica Toma

**Affiliations:** 1 Faculty of Dental Medicine, “Grigore T. Popa” University of Medicine and Pharmacy, 700115 Iasi, Romania; 2 CMI “Dr. Flondor Catalina”, Cajvana, 727100 Suceava, Romania

**Keywords:** nutritional biomarkers, oral candidiasis, pediatric population, vitamin deficiency

## Abstract

**Background/Objectives**: Oral candidiasis is a prevalent fungal infection in young children, often associated with underlying factors such as immunosuppression, poor oral hygiene, or nutritional deficiency. This study aimed to investigate the relationship between nutritional biochemical markers and the presence and severity of oral candidiasis in children aged 6 months to six years. **Methods**: A total of 60 participants were enrolled in a cross-sectional observational study, equally divided into a case group with clinically diagnosed oral candidiasis and a control group without fungal infection. Serum levels of vitamin D, iron, zinc, albumin, and vitamin A were measured in all participants. The severity of candidiasis was assessed using a standardized clinical scoring system. **Results**: Statistical analysis revealed that children with candidiasis exhibited significantly lower levels of all measured biochemical markers than healthy controls. However, no significant correlations were found between the severity of candidiasis and individual nutritional parameters. **Conclusions**: These findings suggest that even moderate deficiencies in essential nutrients may increase susceptibility to oral fungal infections, although they may not directly influence disease severity.

## 1. Introduction

Oral candidiasis, also known as thrush, is a prevalent opportunistic fungal infection, particularly in pediatric populations, with a higher prevalence observed among infants and young children [1]. This condition is predominantly instigated by the organism *Candida albicans*, a species classified as a commensal inhabitant of the oral cavity, which typically coexists harmlessly within the human microbiome [2]. However, this organism may shift to a pathogenic role under specific conditions, including, but not limited to, immunosuppression resulting from various medical conditions or treatments, inadequate oral hygiene practices, and nutritional deficiencies that compromise the host’s immune defenses [3].

In the context of healthy children, oral candidiasis is often self-limiting [4]. Nonetheless, it is important to note that in pediatric patients with certain underlying risk factors, such as those undergoing immunosuppressive therapies, individuals with chronic systemic diseases, or those with significant nutritional deficits, oral candidiasis can present as recurrent or evolve into a more severe clinical manifestation [5]. Such cases necessitate closer monitoring and targeted therapeutic interventions to reduce complications and ensure adequate infection management.

Nutrition is essential in maintaining optimal immune function and ensuring the structural integrity of the mucosal barrier [6]. The mucosal barrier serves as a first line of defense against pathogens, and the nutritional status of an individual significantly influences its efficacy. Deficiencies in essential micronutrients, including but not limited to vitamin D, vitamin A, zinc, and iron, have been extensively studied and found to correlate with an increased susceptibility to a diverse range of infections [7]. For instance, specific deficiencies can lead to conditions such as mucocutaneous candidiasis, which should be a point of concern in populations with inadequate micronutrient intake [8]. Furthermore, protein-energy malnutrition, often indicated by reduced serum albumin levels, poses an additional risk to host defenses [9]. Such malnutrition not only weakens the immune response but also heightens vulnerability to opportunistic infections [10], particularly in vulnerable populations such as children. The relationship between nutrition and immune function highlights the importance of maintaining adequate dietary intake to support overall health and effectively combat infectious diseases.

Despite the well-documented role of nutrition in modulating immune function and maintaining mucosal integrity, few studies have specifically explored the biochemical nutritional profile of children with oral candidiasis in non-hospitalized, otherwise healthy pediatric populations. Existing literature has primarily focused on immunocompromised patients or those with underlying systemic conditions [3,11,12], leaving a significant gap in understanding how moderate, subclinical nutritional deficiencies may predispose immunocompetent children to fungal infections of the oral mucosa. Furthermore, data on region-specific pediatric populations, such as those from Northeastern Romania, where nutritional disparities and access to preventive healthcare may vary, are particularly scarce.

In Romania, nutritional deficiencies persist as a significant pediatric health issue, particularly in the northeastern regions, where socioeconomic and healthcare disparities are more pronounced [13]. National and regional surveillance data have highlighted an elevated prevalence of suboptimal serum levels of iron, vitamin D, and vitamin A among preschool-aged children, often in the absence of overt clinical signs [14].

Oral candidiasis, while frequently observed in pediatric practice, is underrepresented in local epidemiological reporting, despite its recognized association with mucosal immune compromise. Conducting this investigation within the Romanian context provides insight into how nutritional disparities may influence infection risk in underrepresented populations. It is important to note that “nutritional deficiency” refers specifically to the inadequate availability of one or more essential nutrients, whereas “malnutrition” encompasses a broader spectrum of nutritional imbalances, including both deficiencies and excesses.

The biomarkers selected in this study—vitamin D, iron, zinc, albumin, and vitamin A—were chosen for their relevance to immune modulation and mucosal barrier integrity [15]. While these markers provide a representative assessment of nutritional status in this age group, it is acknowledged that other parameters, including folate or B-complex vitamins, may also play a contributory role [16] and warrant further investigation.

This study investigated whether children from Northeastern Romania with oral candidiasis exhibit distinct nutritional profiles compared to their healthy counterparts. Additionally, the research aims to determine whether the severity of candidiasis is correlated with serum concentrations of essential nutrients. The null hypothesis formulated for this study posits no significant difference in the levels of nutritional biochemical markers (vitamin D, iron, zinc, albumin, and vitamin A) between children afflicted with oral candidiasis and their healthy controls, as well as no correlation between the serum levels of nutritional markers and the severity of candidiasis.

## 2. Materials and Methods

### 2.1. Study Population

This cross-sectional, observational study investigated the association between nutritional biochemical status and oral candidiasis in children. The protocol was approved by the “Grigore T. Popa” University Ethics Committee (Ethical approval number 390/30 January 2024), and informed consent was obtained from the parents or legal guardians of all participants.

Participants were recruited from two pediatric dental clinics: the Pediatric Dentistry Clinic of “Grigore T. Popa” University of Medicine and Pharmacy in Iași and a private practice in Cajvana, Suceava, both located in northwestern Romania. A total of 60 children aged between 6 months and 6 years were included, divided into two groups: a case group consisting of 30 children diagnosed with oral candidiasis and a control group of 30 healthy children without clinical evidence of fungal infection. No formal matching was employed between the case and control groups. Identical inclusion and exclusion criteria were applied across both recruitment sites to ensure demographic comparability and reduce the risk of selection bias.

The study included children aged 6 to 71 months to capture the early developmental window during which both oral candidiasis and nutritional deficiencies are most prevalent [17]. The lower age limit of 6 months was selected to exclude neonatal factors and to align with the introduction of complementary feeding, a critical period for micronutrient status. The upper limit of 6 years reflects the typical transition to school age, when dietary patterns and oral microbiota become more stable and less susceptible to early-life nutritional influences [18].

The following exclusion criteria were applied: known primary or secondary immunodeficiencies (e.g., congenital immunodeficiencies, HIV infection); chronic systemic illnesses (e.g., diabetes mellitus, renal failure, liver disease, cancer); diagnosed autoimmune disorders; genetic syndromes affecting growth, development, or immunity; severe malnutrition requiring therapeutic feeding protocols; current diagnosis or history of oral mucosal diseases unrelated to candidiasis (e.g., lichen planus, herpetic stomatitis); diagnosed severe anemia (e.g., hemoglobin < 8 g/dL); the use of systemic or topical antifungal agents, systemic corticosteroids, or immunosuppressive therapy within the last 30 days; antibiotic use in the previous 14 days; ongoing vitamin or micronutrient supplementation (e.g., high-dose vitamin D or iron therapy); the presence of fixed dental appliances or prosthetics; ongoing active dental caries treatment; recent dental surgery or oral trauma (within the past 2 weeks); children on special diets (e.g., ketogenic, dairy-free, gluten-free), not representative of the general population; and the current use of oral probiotics.

Children undergoing conservative dental treatment (e.g., cavity preparation, temporary restorations) or recent oral surgery (within the past two weeks) were excluded to minimize local factors that could alter mucosal integrity, introduce antiseptic agents, or induce inflammation that might independently influence the risk or appearance of oral candidiasis. Exclusion was limited to oral procedures; children with a history of systemic surgery were not excluded unless they met other criteria related to immune status or recent medication use.

### 2.2. Clinical Evaluation

The diagnosis of oral candidiasis was based on clinical criteria, including the presence of white, curd-like plaques on the oral mucosa that could be partially removed, leaving behind erythematous mucosa. Clinical evaluation included a structured oral examination. To evaluate the severity of oral candidiasis in the pediatric population, a structured clinical scoring system was applied, incorporating five key domains: lesion extent, number of oral sites affected, lesion consistency, reported discomfort during feeding, and duration of symptoms. Each domain was graded on a 4-point scale (0–3), allowing for a maximum cumulative score of 15 points (Table 1). This scoring framework was developed by adapting established principles from clinical mucosal assessment tools, such as the Oral Mucositis Assessment Scale (OMAS) [19]. It was customized to reflect the characteristic features of candidiasis in young children. Although this particular model has not yet undergone formal validation, it was designed to support consistent and reproducible severity classification within the context of this observational study.

All clinical examinations were conducted by pediatric dentists who had been trained and calibrated in advance using a shared diagnostic protocol. Oral candidiasis was diagnosed based on consistent clinical criteria—namely, the presence of white, partially removable plaques on the oral mucosa with associated erythema. To ensure diagnostic consistency across sites, a standardized scoring system was used for assessing severity, and inter-examiner agreement was verified prior to the start of the study.

### 2.3. Nutritional Biochemical Markers

Venous blood samples were collected on the same day as the clinical examination, between 8:00 and 10:30 a.m., following a minimum 6 h fasting period. Blood draws were performed at the affiliated laboratory by trained personnel under standardized conditions to reduce biological variability related to circadian rhythm or recent food intake.

The following parameters were measured: serum vitamin D (ng/mL), serum iron (µg/dL), serum zinc (µg/dL), serum albumin (g/dL), and serum vitamin A (µg/dL). All assays were conducted with standard automated biochemical methods, and internal quality control procedures were implemented.

### 2.4. Statistical Analysis

A power analysis was conducted to estimate the sample size required to detect statistically significant differences in nutritional biochemical markers between children with oral candidiasis and healthy controls. Using the software G*Power 3.1, a two-tailed *t*-test for independent samples was selected, assuming the following: effect size (Cohen’s d): 0.7 (moderate-to-large, based on pilot data and relevant literature); alpha level (α): 0.05; power (1 − β): 0.80 (80%); allocation ratio (n2/n1): 1 (equal group sizes). The effect size estimate was informed by pilot data and supported by prior literature, which showed substantial differences in micronutrient levels between pediatric populations with and without mucosal infections [20].

The analysis indicated that a minimum of 26 subjects per group would be required to detect significant differences in nutritional parameters between groups. To account for potential dropouts and variability and to enhance the robustness of subgroup analyses, a total of 60 participants (30 per group) were enrolled. With 30 participants per group, the study exceeded the required sample size. Post hoc power analysis indicated that the final sample achieved statistical power greater than 0.90 for primary nutritional comparisons, with observed effect sizes ranging from 0.65 to 0.85 across measured biomarkers.

Descriptive statistics were calculated for all demographic and clinical variables. Continuous variables were expressed as means and standard deviations (SD), while categorical variables were summarized as counts and percentages. The distribution of the severity score was evaluated using the Shapiro–Wilk test and the visual inspection of histograms. Independent-samples *t*-tests were used to compare nutritional biomarkers between groups (case vs. control). Categorical comparisons (e.g., sex distribution) were assessed using chi-square tests.

To explore associations between nutritional status and clinical severity, Pearson correlation coefficients were initially computed between each biochemical marker and the severity score. Subsequently, simple linear regression analyses were performed using the severity score as a continuous outcome variable and each nutritional biomarker as an independent predictor. Model performance was assessed using standardized beta coefficients, R^2^ values, and associated *p*-values. Scatter plots with fitted regression lines were generated to visualize these relationships.

All analyses were conducted using IBM SPSS Statistics, version 27.0 (IBM Corp., Armonk, NY, USA), and G*Power 3.1 was used for power calculations. A *p*-value < 0.05 was considered statistically significant.

This study adheres to the STROBE guidelines for cross-sectional studies. A completed checklist is provided in the Supplementary Materials [Supplementary File S1].

## 3. Results

### 3.1. Demographic Data

A total of 60 children from the Northeast of Romania were enrolled in the study, with 30 allocated to the case group (children diagnosed with oral candidiasis) and 30 to the control group (healthy children without signs of fungal infection). All enrolled participants completed the study, and no drop-outs were recorded during data collection.

The two groups were similar regarding age and gender distribution; the mean age for the case group was 34.2 ± 16.4 months (6–71 months), and for the control group, it was 33.5 ± 15.9 months (6–70 months) (Table 2).

### 3.2. Nutritional Markers

The comparative analysis of nutritional biochemical parameters between the case group and the control group revealed statistically significant differences across all measured markers. Children in the case group consistently exhibited lower mean levels of vitamin D, serum iron, zinc, albumin, and vitamin A. These differences were statistically significant, with *p*-values well below the 0.0001 threshold and high t-statistics (Table 3).

The mean serum vitamin D level in the case group was 20.2 ng/mL (±2.9), markedly lower than the control group’s mean of 25.0 ng/mL (±2.9). Similar trends were noted for iron (60.6 ± 7.8 µg/dL vs. 76.0 ± 7.4 µg/dL) and zinc (68.7 ± 7.4 µg/dL vs. 81.2 ± 7.6 µg/dL). The case group also exhibited reduced protein status, as indicated by a mean albumin level of 3.39 ± 0.22 g/dL, compared to 3.78 ± 0.21 g/dL in the control group. Vitamin A was similarly lower in the affected group (29.2 ± 3.8 µg/dL vs. 35.6 ± 4.3 µg/dL). Each difference between groups was statistically significant (*p* < 0.05), indicating a consistent trend of nutritional deficiency in children with oral candidiasis.

### 3.3. Severity Score Distribution

**Figure 1:**
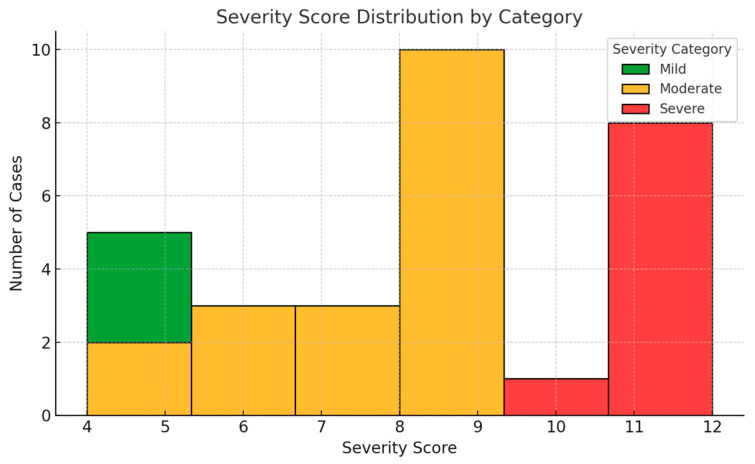
Distribution of oral candidiasis severity scores among affected children. Most cases fell within the moderate range (5–9 points). The score distribution approximated normality and was used as a continuous variable in subsequent analyses.

Among the children diagnosed with oral candidiasis, severity scores ranged from 4 to 12, with a mean value of 7.3 (±2.5). The distribution of scores demonstrated approximate normality, as assessed both visually and by the Shapiro–Wilk test (*p* = 0.162). The majority of cases (60%) fell within the moderate range (5–9 points), while 23.3% were classified as mild (0–4 points) and 16.7% as severe (10–15 points) (Figure 1). No statistically significant differences in severity scores were observed based on sex (*p* = 0.44) or age category (children under 3 years versus those aged 3–6 years; *p* = 0.52).

The scoring system was developed to capture both the clinical presentation and functional impact of the condition. Its distribution in this sample suggests an ability to differentiate disease intensity within an outpatient pediatric population. For subsequent analysis, the total severity score was treated as a continuous variable to assess its relationship with individual biochemical markers of nutritional status.

### 3.4. Correlation Between Nutritional Markers and Severity of Candidiasis

Correlation analyses were conducted within the case group using Pearson correlation coefficients to evaluate the potential influence of nutritional status on the clinical severity of oral candidiasis. The severity score, derived from a structured clinical assessment scale (range 0–15), was compared to each biochemical parameter.

None of the individual biochemical parameters showed a statistically significant correlation with the severity score (*p* > 0.05 for all comparisons) (Table 4). This suggests that while lower nutritional status is associated with the presence of candidiasis, it may not directly influence the extent or intensity of mucosal involvement once infection is established.

### 3.5. Regression Analysis of Biomarkers and Severity Score

To explore potential relationships between nutritional status and clinical severity, linear regression analyses were conducted using the candidiasis severity score as a continuous outcome measure. Each nutritional biomarker was evaluated individually as a predictor in separate models, allowing for the assessment of direct associations within the affected pediatric subgroup.

The results of these analyses did not reveal any statistically significant associations. For vitamin D, the regression coefficient was –0.126 (R^2^ = 0.054, *p* = 0.192); for serum iron, −0.049 (R^2^ = 0.005, *p* = 0.667); for zinc, −0.111 (R^2^ = 0.034, *p* = 0.284); for albumin, −0.202 (R^2^ = 0.045, *p* = 0.229); and for vitamin A, −0.192 (R^2^ = 0.045, *p* = 0.232). These findings suggest that the severity of mucosal involvement, as reflected in the clinical scoring system, is not linearly related to the measured levels of these micronutrients.

**Figure 2:**
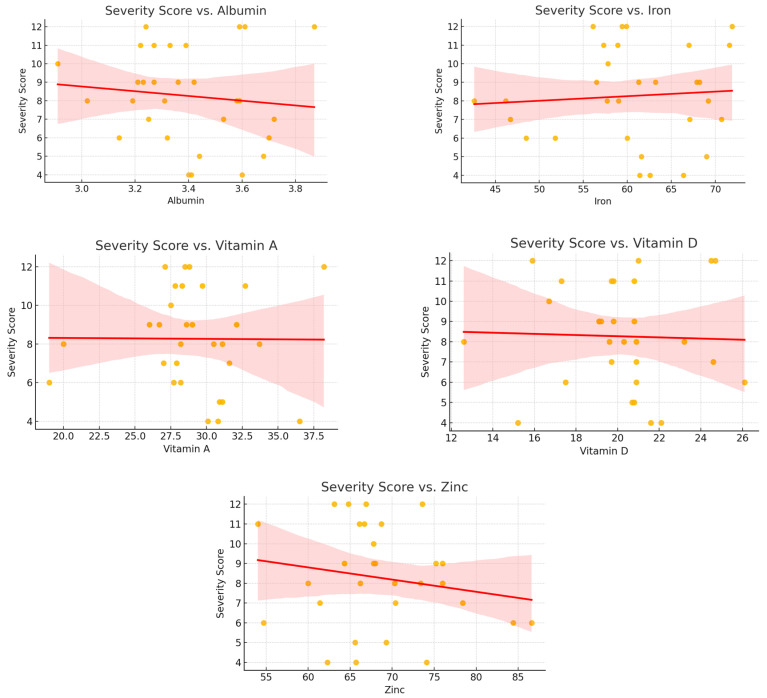
Scatter plots showing the relationship between nutritional biomarkers and candidiasis severity scores in the case group. No significant trends were observed.

Scatter plots with fitted regression lines are presented in Figure 2, illustrating the absence of clear trends between biomarker concentrations and severity scores.

## 4. Discussion

The findings of this study demonstrate a clear and statistically significant association between oral candidiasis and reduced levels of key nutritional biomarkers in children aged six months to six years from northeastern Romania. Despite intentionally maintaining moderate differences between the case and control groups, significant reductions were observed in serum vitamin D, iron, zinc, albumin, and vitamin A in children diagnosed with candidiasis.

Among the micronutrients evaluated, vitamin D emerges as particularly relevant due to its well-established immunomodulatory functions [21]. In addition to its classical role in calcium homeostasis and bone metabolism [22], vitamin D plays an integral part in regulating both innate and adaptive immune responses [23]. It promotes the synthesis of antimicrobial peptides, such as cathelicidin and defensins [24,25], which are essential for epithelial defense mechanisms. Additionally, it modulates the activity of macrophages and dendritic cells, thereby influencing antigen presentation and cytokine production [26].

Deficiency in vitamin D has been associated with impaired mucosal immunity, reduced epithelial barrier integrity, and diminished phagocytic activity [27], all of which may contribute to increased vulnerability to opportunistic pathogens such as *C. albicans*. Observational data have linked low vitamin D levels with an increased risk of oral candidiasis in HIV-positive populations [28], though pediatric data remain limited.

In pediatric populations, where immune systems are still maturing and micronutrient reserves can be easily depleted [29], even moderate insufficiency in vitamin D levels may disrupt oral mucosal homeostasis, facilitating fungal colonization and overgrowth [30]. These mechanisms underscore the biological plausibility of the observed association between lower serum vitamin D concentrations and the presence of oral candidiasis in the study cohort.

Iron and zinc are indispensable trace elements that serve as cofactors in numerous enzymatic processes critical to maintaining immune competence and epithelial barrier integrity. Iron is essential for the proliferation and maturation of immune cells, particularly lymphocytes, and plays a central role in the generation of reactive oxygen species used by phagocytes to eliminate invading pathogens [31]. Likewise, zinc is crucial for the structural stability and function of numerous transcription factors [32], and its deficiency has been shown to impair the activity of natural killer cells, neutrophils, and the balance of pro- and anti-inflammatory cytokine responses [33]. Both micronutrients are involved in tissue repair processes, including epithelial regeneration and wound healing [34], which are vital for maintaining an intact mucosal surface.

Both clinical and experimental studies have documented that suboptimal levels of iron and zinc are associated with increased susceptibility to bacterial, viral, and fungal infections, particularly in pediatric populations with heightened nutritional demands [35,36,37]. For instance, Black [38] reported that zinc deficiency contributes to diminished epithelial barrier function and increased susceptibility to infection in children, while Mantadakis et al. observed similar associations with iron deficiency [39].

In the context of oral health, deficiencies in these micronutrients may compromise local immune surveillance and epithelial integrity, thereby facilitating the colonization and subsequent overgrowth of opportunistic organisms such as *Candida albicans*. The significantly lower serum concentrations of iron and zinc observed in the present study among children with candidiasis support this pathophysiological model, underscoring the need to consider micronutrient assessments as part of a broader preventive strategy in pediatric oral healthcare.

Serum albumin, a major plasma protein synthesized by the liver, serves as a widely recognized marker of protein-energy status and overall nutritional adequacy [40]. In pediatric populations, hypoalbuminemia may indicate insufficient dietary protein intake, impaired hepatic synthetic function, or increased protein catabolism secondary to inflammation or infection [41]. Beyond its nutritional implications, low serum albumin has also been linked to altered colloid osmotic pressure, reduced antioxidant capacity, and impaired micronutrient transport—factors that can collectively compromise immune resilience [42]. The consistently lower albumin levels observed among children with oral candidiasis in this study may therefore reflect a combination of systemic nutritional insufficiency and heightened metabolic demands associated with subclinical inflammatory processes.

Vitamin A plays a distinct yet complementary role in mucosal defense through its effects on epithelial cell differentiation, barrier maintenance, and immune modulation [43]. It is essential for the generation of retinoic acid, which regulates the homing of lymphocytes to mucosal sites and enhances the production of secretory immunoglobulin A (sIgA), a critical component of localized antimicrobial defense [44]. In the absence of adequate vitamin A, epithelial surfaces become more susceptible to colonization and invasion by pathogens, ref. [45] including fungal organisms such as *C. albicans*. The significantly reduced serum levels of vitamin A found in the candidiasis group further support the hypothesis that micronutrient deficiencies are associated with diminished mucosal immunity and heightened susceptibility to opportunistic infections in early childhood.

While limited pediatric data exist on the association between nutritional biomarkers and oral candidiasis, several international studies provide valuable context. In a study of Mexican preschoolers, Stecksén-Blicks et al. [1] found no direct link between Candida colonization and micronutrient levels; however, they identified high sugar intake as a key risk factor. This highlights the multifactorial nature of oral fungal overgrowth, where dietary composition—not only nutrient adequacy—may shape the oral environment. Similarly, Yahya et al. [46] reported elevated C. albicans loads and greater caries severity among obese children, suggesting that both over- and undernutrition may compromise oral microbial balance. Hernawati [47] documented a pediatric case where vitamin deficiencies coincided with poor oral hygiene in a child with candidiasis, reinforcing the interplay between systemic and local factors. A cross-sectional study conducted by Thekiso et al. (2018) [48] among adults with tuberculosis found that individuals presenting with deficiencies in zinc, vitamin A, and vitamin D were more likely to exhibit clinical signs of oral candidiasis. Although that study involved a predominantly immunocompromised adult cohort, the convergence in implicated micronutrients suggests a broader relevance of nutritional status in modulating mucosal defense mechanisms. The overlap between those findings and the results observed in the present pediatric sample supports the hypothesis that deficits in key micronutrients may compromise epithelial integrity and immune responsiveness, thereby facilitating fungal overgrowth across distinct clinical contexts.

Notably, few studies have specifically examined the relationship between micronutrient levels and the severity of oral candidiasis. Our results align with existing literature in showing that children with candidiasis exhibit significantly lower levels of several key nutrients. However, the absence of strong associations between individual biomarkers and severity scores in our data suggests that, once infection is established, local factors may play a more prominent role in modulating clinical expression. This distinction may explain the apparent gap in the literature linking nutrient status to symptom intensity, rather than susceptibility alone. This finding suggests that, although nutritional deficiencies may be associated with an increased predisposition for children to the development of candidiasis, the extent of clinical manifestation may be more strongly influenced by local oral environmental factors. Variables such as oral hygiene practices, salivary flow rate and composition, microbial biofilm complexity, and mucosal pH are likely to play a key role in modulating the progression and severity of the infection once colonization by Candida species has occurred [49].

While the current study focused on a core set of micronutrients and protein markers with established roles in immune modulation, it is likely that other nutritional biomarkers also contribute to host susceptibility to candidiasis. Folate and vitamin B12, for example, are essential for cellular proliferation and epithelial regeneration [50], and deficiencies in these vitamins may impair mucosal repair mechanisms. Likewise, trace elements such as selenium and copper have been implicated in both antioxidant defense and immune cell function [51,52] and may influence the mucosal response to fungal pathogens. Emerging literature also suggests that deficiencies in polyunsaturated fatty acids and vitamin E could alter local inflammatory responses and epithelial barrier integrity [53]. These biomarkers, which were not assessed in the present study, represent plausible mediators of both nutritional status and candidiasis risk and warrant investigation in future studies employing broader biochemical panels.

Differences in oral cavity conditions between affected and unaffected children may also reflect underlying differences in nutritional status. While this study focused on specific biochemical indicators of micronutrient deficiency, broader forms of malnutrition, including protein-energy malnutrition, may also further influence oral health through their effects on salivary composition, mucosal pH, and epithelial turnover. For example, reduced salivary flow and diminished levels of antimicrobial peptides have been observed in malnourished children [54], potentially facilitating the colonization and persistence of Candida species. Additionally, chronic undernutrition may impair tissue repair and barrier function [55], increasing the oral mucosa’s vulnerability to opportunistic infections.

As this was a cross-sectional study, the directionality of the observed associations cannot be established. While lower levels of several key micronutrients were identified in children with oral candidiasis, these findings should be interpreted as associative rather than causal. The possibility that candidiasis or related factors may influence nutritional status, or that both are affected by shared confounders, cannot be excluded. Longitudinal or interventional studies are needed to clarify temporal relationships and causal mechanisms.

One notable limitation of the present study is the use of a non-validated severity scoring tool. The proposed severity scoring system combines clinician-assessed parameters (lesion extent, number of affected areas, lesion consistency) with caregiver-reported features (duration of symptoms and feeding-related discomfort). While this approach offers a more holistic view of disease impact in young children, it also introduces potential variability, especially for behaviorally influenced parameters. These may be subject to caregiver interpretation and influenced by a child’s temperament, feeding behavior, or emotional state. Despite this limitation, the inclusion of these domains was intended to reflect functional burden and symptomatic duration, which are clinically relevant in pediatric contexts. The model should be viewed as a preliminary framework that warrants further refinement and validation in future studies, ideally through inter-rater agreement testing and comparison with microbiological or treatment-response outcomes.

This study did not include direct assessments of dietary intake or detailed socioeconomic indicators, both of which may influence nutritional status and, by extension, susceptibility to oral candidiasis. Variations in food access, parental education, hygiene practices, and healthcare utilization are all social determinants of health that could contribute to disparities in nutritional biomarkers and oral health outcomes. Additionally, while race and ethnicity were not recorded, the study population was drawn from a relatively homogeneous ethnic region in northeastern Romania, which may mitigate—but not eliminate—cultural and genetic variability. These omissions represent potential sources of residual confounding and should be addressed in future studies through the use of structured dietary assessment tools and standardized sociodemographic questionnaires.

Age is a critical determinant of both nutritional needs and immune maturation in early childhood, and it may influence the susceptibility to and severity of mucosal infections. Infants and toddlers undergo rapid growth phases that increase micronutrient demands [56], while oral microbiota composition and salivary function evolve significantly over time. Although the case and control groups in this study were similar in age distribution, subtle age-related differences in micronutrient status may still have influenced individual susceptibility or recovery. Similarly, although no significant differences in severity were observed by sex, it is acknowledged that hormonal, immunological, and behavioral differences between males and females may emerge as early as in early childhood. These variables should be considered in future studies using multivariate models to adjust for potential confounding effects.

Another limitation of the study is the absence of follow-up blood samples following clinical recovery. Although symptom duration was recorded and caregivers were encouraged to return for reassessment, the study was designed as a single-time-point, cross-sectional analysis. Logistical and ethical considerations related to repeated blood sampling in a pediatric population precluded the longitudinal monitoring of nutrients. Future studies could benefit from incorporating serial measurements of serum biomarkers to assess whether observed deficiencies persist, resolve with treatment, or fluctuate in relation to disease activity.

Future research should consider longitudinal designs to explore causal pathways and assess the potential benefits of targeted nutritional interventions for the prevention or treatment of oral candidiasis. Additionally, integrating immunological assays, inflammatory markers, and oral microbiota profiling would offer a more comprehensive understanding of the complex interplay between nutrition, mucosal immunity, and microbial ecology in pediatric populations.

## 5. Conclusions

Children diagnosed with candidiasis consistently exhibited lower serum levels of vitamin D, iron, zinc, albumin, and vitamin A compared to their healthy peers. These findings suggest that even moderate nutritional deficiencies may contribute to the pathogenesis of oral fungal infections in pediatric populations.

While the study did not find a direct correlation between disease severity and individual nutrient levels, the consistent pattern of lower biomarker values in the case group underscores the importance of early nutritional screening as a preventive tool. Ensuring the adequate intake and monitoring of key micronutrients may represent an accessible and cost-effective strategy for reducing the risk of opportunistic infections in childhood.

## Figures and Tables

**Table 1:** Clinical scoring system for oral candidiasis.

Criterion	0 Points	1 Point	2 Points	3 Points
Lesion extent	No lesions	≤25% of oral mucosa	25–50%	>50% or diffuse
Number of areas affected	None	1 area (e.g., tongue)	2 areas	≥3 areas
Lesion consistency	N/A	Easily detachable, thin	Thick, adherent	With erosions/bleeding
Pain/Discomfort	Absent	Mild	Discomfort during feeding	Refusal to feed/crying when nursing
Duration of symptoms	<2 days	2–4 days	5–7 days	>7 days
Interpretation of the Total Score (maximum 15 points): 0–4 points: Mild candidiasis 5–9 points: Moderate candidiasis 10–15 points: Severe candidiasis				

**Table 2:** The demographic characteristics for the study groups.

Variable	Case Group (n = 30)	Control Group (n = 30)
Number of participants	30	30
Mean age (months) ± standard deviation	34.2 ± 16.4	33.5 ± 15.9
Age range (months)	6–71	6–70
Male [n (%)]	16 (53.3%)	17 (56.7%)
Female [n (%)]	14 (46.7%)	13 (43.3%)

**Table 3:** The biochemical parameters for the study groups.

Parameter	Case Group (Mean ± SD)	Control Group (Mean ± SD)	t-Statistic	*p*-Value
Vitamin D (ng/mL)	20.2 ± 2.9	25.0 ± 2.9	6.41	<0.0001
Serum Iron (µg/dL)	60.6 ± 7.8	76.0 ± 7.4	7.97	<0.0001
Zinc (µg/dL)	68.7 ± 7.4	81.2 ± 7.6	6.63	<0.0001
Albumin (g/dL)	3.39 ± 0.22	3.78 ± 0.21	7.41	<0.0001
Vitamin A (µg/dL)	29.2 ± 3.8	35.6 ± 4.3	6.56	<0.0001

**Table 4:** Correlation between nutritional markers and severity of candidiasis.

Parameter	Correlation Coefficient (r)	*p*-Value
Vitamin D (ng/mL)	−0.03	0.8629
Serum Iron (µg/dL)	0.08	0.6933
Zinc (µg/dL)	−0.18	0.3439
Albumin (g/dL)	−0.11	0.5615
Vitamin A (µg/dL)	−0.01	0.9701

## Data Availability

The original contributions presented in this study are included in the article. Further inquiries can be directed to the corresponding author.

## Supplementary Materials

The following supporting information can be downloaded at: https://www.mdpi.com/article/10.3390/nu17111815/s1, File S1: STROBE Statement—Checklist of items that should be included in reports of cross-sectional studies.

## Abbreviations

The following abbreviations are used in this manuscript:

HIV	Human Immunodeficiency Virus
OMAS	Oral Mucositis Assessment Scale
SD	Standard Deviation
sIgA	Secretory immunoglobulin A
